# Comparison of postoperative complication between Laryngeal Mask Airway and endotracheal tube during low-flow anesthesia with controlled ventilation

**DOI:** 10.12669/pjms.292.2980

**Published:** 2013-04

**Authors:** Ali Peirovifar, Mahmood Eydi, Mir Mousa Mirinejhad, Ata Mahmoodpoor, Afsaneh Mohammadi, Samad EJ Golzari

**Affiliations:** 1 Ali Peirovifar, Associate Professor of Anesthesiology, Fellowship of Critical Care Medicine, Faculty of Medicine, Anesthesiology Research Team, Department of Anesthesiology, Tabriz University of Medical Sciences, Tabriz, Iran.; 2 Mahmood Eydi, Associate Professor of Anesthesiology, Faculty of Medicine, Anesthesiology Research Team, Department of Anesthesiology, Tabriz University of Medical Sciences, Tabriz, Iran.; 3 Mir Mousa Mirinejhad, Associate Professor of Anesthesiology, Fellowship of cardiovascular anesthesia, Faculty of Medicine, Anesthesiology Research Team, Department of Anesthesiology, Tabriz University of Medical Sciences, Tabriz, Iran.; 4 Ata Mahmoodpoor, Assistant Professor of Anesthesiology, Fellowship of Critical Care Medicine, Faculty of Medicine, Anesthesiology Research Team, Department of Anesthesiology, Tabriz University of Medical Sciences, Tabriz, Iran.; 5 Afsaneh Mohammadi, Medicine student, Faculty of Medicine, Anesthesiology Research Team, Department of Anesthesiology, Tabriz University of Medical Sciences, Tabriz, Iran.; 6 Samad EJ Golzari, Physical Medicine and Rehabilitation Research Center, Students’ Research Committee, Anesthesiology Research Team, Department of Anesthesiology, Tabriz University of Medical Sciences, Tabriz, Iran.

**Keywords:** Laryngeal Mask Aiway, Endotracheal Tube, Low-flow anesthesia, Complications, Controlled ventilation

## Abstract

***Objective: ***To compare the postoperative complications between Laryngeal Mask Airway (LMA) and endotracheal tube (ETT) during low-flow anesthesia with controlled ventilation.

***Methodology: *** Eighty adult Patients with ASA class I or II were randomly allocated into two forty-patient groups (ETT or LMA). Cuff pressure was monitored during anesthesia. After high uptake period, fresh gas flow (FGF) was decreased to 1 lit/min and isoflurane set to 1%. Monitoring during anesthesia included non-invasive blood pressure, ECG, ETCO_2_ and pulse oximetry. System leakage (>100 ml/min), rebreathing and any attempt to increase FGF to overcome the leak were monitored during anesthesia. Later, patients were extubated and transferred to Post Anesthesia Care Unit (PACU). In PACU, the incidence of sore throat, cough, difficulty in swallowing and shivering was monitored for all patients.

***Results: ***Leakage was observed in two and three cases in ETT and LMA groups respectively (*P*>0.05). Postoperative cough, sore throat and difficulty in swallowing were significantly less in LMA than ETT group. No significant difference was observed regarding ETCo2 values between 2 groups.

***Conclusion:*** If careful measures regarding insertion techniques, correct LMA position and routine monitoring of LMA cuff pressure are taken, LMA can be used as a safe alternative with lower incidence of post operation complication compared with ETT during low-flow controlled anesthesia with modern anesthetic machines.

## INTRODUCTION

Low-flow anesthesia has many advantages in term of decreasing atmospheric pollution, economy and better maintenance of airway temperature and humidification.^1^^,^^2^ Baker in 1994 used following classification for low-flow anesthesia: minimal flow as <500ml fresh gas flow (FGF) per minute, low-flow as > 0.5-1 lit/min, medium flow as 2-4 lit/min of FGF and very high-flow as more than 4 lit/min of FGF.^3^ However, an appropriate sealing is necessary for low-flow anesthesia especially during controlled ventilation.^4^

Laryngeal Mask Airway (LMA) is regarded as a safer supraglottic airway for general anesthesia compared with endotracheal tubes (ETTs) having an established role on difficult airway and spontaneous ventilation.^5^^-^^7^ Although the LMA does not provide a watertight seal, it has been used largely during positive pressure ventilation in adults and children.^8^^,^^9^ Some studies support the concept of safety of using LMA during low-flow anesthesia.^10^^,^^11^

Postoperative complications after ETT and LMA are common; however, some studies have shown that the incidence of complications like sore throat following ETT usage is much higher than LMA.^12^ There are numerous case reports on the complications of LMA like sore throat, hoarseness, bleeding and nerve injury.^13^^-^^15^ The most important possible mechanism is high cuff pressure with N_2_0 usage during maintenance of anesthesia.^16^^,^^17^ Considering the fact that the rate of postoperative complications with ETT is likely to be more than LMA and using LMA during low-flow controlled anesthesia needs tight sealing of airway that needs requiring appropriate inflation of LMA cuff and on the other hand knowing that LMA cuff pressure correlates with sore throat and complications, we decided to compare the postoperative complication between LMA and ETT during low-flow anesthesia with controlled ventilation.

## METHODOLOGY

After approval of ethics committee of Tabriz University of Medical Sciences, 80 adult patients who were scheduled to undergo elective ophthalmic surgeries with duration of almost one hour were enrolled in this randomized clinical trial. Randomization was performed with Grav O tron 2.0 (http://3d2f.com/tags/randomization).

A power analysis was performed to determine the number of patients needed to detect a 50% difference in the post operative complication between two devices based on previously published study by El-Sefy et al. An alpha error of 0.05 and power of 85% was used for this calculation. As we expected failure to follow up during the study we increased the number of patients in each group to 40. This trial is registered with IRCT registry ID: IRCT201203042582N5. Exclusion criteria consisted of expected difficult airway, history of sore throat or common cold within 10 previous days, known allergy to latex and not being fasted. Patients with ASA class I or II were randomly allocated to two forty patient groups (ETT or LMA). All patients received 2 mg midazolam for premedication. Later, induction of anesthesia was performed with propofol 2mg/kg (Diprivan, Astra-Zenca), Fentanyl 2µg/kg, lidocain 1 mg/kg and atracurium 0.5 mg/kg. Mask ventilation was performed with oxygen 100% for three minutes until achieving suitable condition. In first group, an appropriate size LMA 4 or 5 based on manufacturer recommendation was inserted using standard technique. We instilled isotonic saline over LMA cuffs before insertion. The cuff of LMA then was inflated stepwise until the audible cuff leak decreased. An appropriate size ETT (7.5 for females) and 8 for males was inserted in second group and cuff was inflated until 25 mmHg Cuff pressure was monitored during anesthesia. After satisfactory ventilation with several manual breathes, the airway device was connected to anesthesia machine. Maintenance of anesthesia was performed with O_2_/N_2_O 50%, isoflurane and fresh gas flow of 6 lit/min for 10 minutes to deliver isoflurane and N_2_O during high uptake period after that FGF decreased to 1 lit/min and isoflurane sets to 1%. In case of insufficient anesthesia, 50-100 µg fentanyl Was injected. Ventilation was continued with tidal volume of 8 ml/kg and ventilator frequency was adjusted based on ETCO_2_. Monitoring during anesthesia included non-invasive blood pressure, ECG, ETCO_2_ and pulse oximetry. System leakage (>100 ml/min), rebreathing and any attempt to increase FGF to overcome the leak were monitored during anesthesia. Isoflurane was discontinued 5 minutes to the end of surgery and FGF increased to 6 lit/min and O_2_ to 100% to wash out the anesthetics. Later, patients were extubated and transferred to Post Anesthesia Care Unit (PACU). In PACU the incidence of sore throat, cough, difficulty in swallowing and shivering were monitored for all patients till 2 hours.

All data were analyzed by SPSS version 17. Data were reported as mean±SD. Qualitative and quantitative variables were analyzed with chi square and unpaired t tests respectively. P value less than 0.05 was considered as statistically significant.

## RESULTS

Eighty adult patients (61% female and 31% male) were enrolled in this trial. Demographic characteristic of patients are shown in Table-I, while 37.5% of patients had ASA class I and 63.5% had ASA class II. Leakage was observed in five cases (6.2%), from which two cases were in ETT and three cases in LMA groups (*P*>0.05). Sore throat complication was observed in 40% and 5% of the patients in ETT and LMA groups respectively (*P*<0.01, r=0.419). Postoperative shivering was reported in 27.5% and 25% of patients in LMA and ETT groups (*P*>0.05, r=-0.028). Some patients (6.3%) in ETT group had difficulty in swallowing; however, no similar cases were reported in LMA group. Cough was seen in 22% of all patients (16 and 2 patients in ETT and LMA groups respectively). Postoperative cough and difficulty in swallowing was significantly less in LMA group than ETT group (*P*<0.05, r=0.25). General results are shown in Table-II. ETCO2 values measured in two groups revealed no significant difference (36.6±3.2 in ETT group vs. 37.5±2.4 in LMA group).

**Table-I:** Demographic characteristics of patients

	*LMA*	*ETT*	*Pvalue*
M/F	15/25	18/22	>0.05
ASAI/II	18/22	14/26	>0.05
Age	65.6±11.3	71.3±6.75	>0.05
Anesthesia duration	41±5	37±4	>0.05

P value <0.05 is considered as statistically significant

**Table-II:** Complications of LMA and ETT between two groups

	*LMA*	*ETT*	*P value*
Leak	3	2	0.6
Sore throat	2	16	0.001
Difficulty in swallowing	0	5	0.02
Cough	2	16	0.001
Shivering	11	10	0.7

P value <0.05 is considered as statistically significant

## DISCUSSION

Respiratory complications in the form of laryngospasm or bronchospasm during emergence from anesthesia, or postoperative sore throat and postoperative cough are major areas of concern while choosing a device for pediatric airway management. The etiology of respiratory tract complications in the perioperative period is multifactorial, including improper endotracheal tube size, cuff design, lack of airway humidity, trauma during insertion and suctioning, high anesthetic gas flow rates and manipulation of the airway and adjacent tissues.^18^

Engelhardt et al. showed that pressure controlled ventilation using LMA is an alternative to a cuffed ETT during low-flow circle system anesthesia in children. He concluded that low FGF is unlikely to be achieved consistently using an uncuffed ETT due to a substantial leak.^19^

A meta-analysis was performed on randomized prospective trials comparing the laryngeal mask airway (LMA) with other forms of airway management to determine if the LMA possessed any advantages over ETT or facemask. Advantages of LMA over the ETT included: increased speed and ease of placement by both inexperienced and experienced personnel; improved hemodynamic stability at induction and during emergence; minimal increase in intraocular pressure following insertion; reduced anesthetic requirements for airway tolerance; lower frequency of cough during emergence; improved oxygen saturation during emergence; and lower incidence of sore throat in adults. Disadvantages over the ETT were lower seal pressures and a higher frequency of gastric insufflations.^20^ Ates et al. showed that LMA can be regarded as a safe product for airway maintenance during ophthalmic surgery with a stable circulation and few complications.^21^

Cameron et al. conclude that LMA provides as good a gas tight seal as a ETT and is of benefit in reducing anesthetic gas pollution.^7^ These studies showed that LMA could be used as an alternative device for maintenance of anesthesia during spontaneous ventilation. Wahlen and colleagues showed that clinically undetected LMA malpositioning is a significant risk factor for gastric air insufflation in children between 3 and 11 years, undergoing positive pressure ventilation, especially at inspiratory airway pressures above 17 cmH_2_O.^22^

Gastric insufflation and tight sealing are concerns of LMA during controlled low-flow anesthesia; however, no significant difference was observed between LMA and ETT administration in this regard in our study. This may be due to the fact that during low-flow anesthesia we checked for correct positioning of LMA and monitored airway pressure to be less than 15-20 cmH_2_o; additionally, thanks to the modern anesthesia machines, we had low incidence of gastric insufflation and also minimal air leak due to limited FGF.

Honnemann and coworkers showed that the use of LMA was more likely to be associated with gas leak than the use of ETT; however, if modern anesthesia machines and monitors are used, in 96.7% of the patients managed with LMA, a reduction in the FGF to 0.5 L/min was possible. The incidence of postoperative complaints (coughing, sore throat, and swallowing problems) was higher after the use of ETT^11^, which is similar to our study.

Rieger et al. showed that there is a distinct pattern of laryngo-pharyngeal complaints following the use of the LMA and ETT and with regard to minor laryngo-pharyngeal morbidity, the advantage of the LMA to ETT is questionable.^23^

Yu and coworkers in a study showed that for the patients receiving general anesthesia, the use of the LMA resulted in a statistically and clinically significant lower incidence of laryngospasm during emergence, postoperative hoarseness, and coughing than when using the ETT.^24^

Wrong and colleagues showed that cuff pressure in LMA is closely related to the development of sore throat with higher pressures increasing its likelihood. Hence, cuff pressures should be measured routinely using a manometer to minimize the incidence of sore throat.^25^^,^^26^

Bugard et al. showed that a significant increase in cuff pressure is seen during the first 60 minutes. Three minutes after insertion of the laryngeal mask, cuff pressure can be significantly reduced without any major gas leakage. Postoperative sore throat can be reduced when cuff pressure is continuously monitored and kept on low-pressure values.^16^

Dadmehr et al. showed that there was no significant difference between the LMA and ETT regarding complications (nausea, vomiting, coughing and sore throat) in the first 24 hours following the surgery.^27^

Postoperative shivering between two groups did not show any significant difference, as we used the same anesthetic during similar surgeries with equal duration of anesthesia between two groups which is like to previous studies.^28^ Incidence of postoperative cough and difficulty in swallowing between two groups had a significant difference in favor of LMA group which was similar to the previous studies. It might be due to the more neural defect with ETT compared to LMA.

Incidence of complications after anesthesia with LMA seems to be related to intra-cuff pressure, so in lower pressure we expect to have lower complications; however, during low-flow anesthesia we need higher cuff pressure to achieve tight sealing and avoid any probable air leak. The results obtained from our study showed that opposed to mentioned sentences, complications of LMA during low-flow anesthesia are lower than ETT which may be due to better and careful insertion techniques and careful monitoring of LMA position.

 In conclusion: if we use careful insertion techniques, correct LMA positioning and routine monitoring of LMA cuff pressure, we could use LMA as a safe alternative with lower incidence of post operation complications compared to ETT during low-flow controlled anesthesia with modern anesthetic machines.

***Limitations of the study: ***Our study was a single center study in ophthalmic surgery patients, therefore, larger multi-center studies with larger sample sizes are recommended to show the differences between complications of LMA and ETT during low-flow controlled anesthesia. We evaluated only some of the complications between ETT and LMA and some others like gastric insufflations are not mentioned in this study. We also evaluated the complications for two hours after anesthesia and we didn’t compare complications after two hours.
